# A mouse model of subacute liver failure with ascites induced by step-wise increased doses of (-)-epigallocatechin-3-gallate

**DOI:** 10.1038/s41598-019-54691-0

**Published:** 2019-12-02

**Authors:** Xiaoxiao Wang, Lumin Yang, Jiajia Wang, Yafei Zhang, Ruixia Dong, Ximing Wu, Chung S. Yang, Zhenhua Zhang, Jinsong Zhang

**Affiliations:** 1 State Key Laboratory of Tea Plant Biology and Utilization, School of Tea & Food Science, Anhui Agricultural University, Hefei, Anhui China; 2 Department of Infectious Diseases, The Second Affiliated Hospital, Anhui Medical University, Hefei, Anhui China; 3 School of Pharmacy, Anhui Medical University, Hefei, Anhui P.R. China; 4 Department of Forestry and Technology, Lishui Vocational and Technical College, Lishui, Zhejiang, China; 5 Department of Chemical Biology, Ernest Mario School of Pharmacy, Rutgers, The State University of New Jersey, Piscataway, NJ USA; 6 International Joint Research Laboratory of Tea Chemistry and Health Effects, Anhui Agricultural University, Hefei, Anhui China

**Keywords:** Animal disease models, Disease model

## Abstract

Acute liver failure is divided into hyperacute, acute and subacute liver failure. Ascites is a common complication of subacute liver failure. Although animal models of acute liver failure have been established, the study of the pathogenesis of subacute liver failure with ascites complication is hampered by the lack of experimental animal model. The present study aimed at providing a mouse model of subacute liver failure with ascites complication. Kunming mice were intraperitoneally injected with (-)-epigallocatechin-3-gallate (EGCG), a redox-active polyphenol from green tea, for 32 consecutive days with step-wise increased dosage. The EGCG treatment resulted in liver failure as evidenced by extensive hepatocyte necrosis observed histologically along with significant elevation of serum alanine aminotransferase, aspartate aminotransferase, total bilirubin and direct bilirubin levels as well as significant reduction of serum albumin. Liver fibrosis was not observed by Masson staining and fibrosis-associated proteins were not increased. The mortality was less than 12% and the survival mice developed noticeable ascites. Hepatic thioredoxin and glutathione systems were activated by the EGCG. These adaptive responses might render most mice tolerable to the EGCG treatment. The EGCG treatment significantly up-regulated renal urea transporter A1 and promoted its trafficking to apical membrane. These alterations, known to increase water reabsorption, may be responsible, at least in part, for the formation of the ascites. Overall, the mice treated with gradually elevated doses of EGCG exhibits some of the features observed in patients with subacute liver failure, especially ascites. This mouse model is a useful tool for investigating the pathogenesis of subacute liver failure with ascites complication.

## Introduction

Acute liver failure, characterized by sudden and severe hepatic injury, is a serious clinical syndrome with high mortality. Acute liver failure is divided into three groups based upon the interval between development of jaundice and onset of encephalopathy: hyperacute, acute and subacute liver failure^1^. International Association for the Study of the Liver Subcommittee suggested the use of acute liver failure and subacute liver failure as two distinct entities, but not as subgroups of a syndrome^2,3^. Subacute hepatic failure, also known as subfulminant liver failure^4,5^ or late onset hepatic failure^6^, could be caused by multiple factors; however, viral and drug-induced hepatitis are the predominant etiology of all cases^4,5,7^. Among drug-induced liver failure in China, the major type of liver failure is subacute liver failure and the predominant etiological factors are antitubercular agent (25.5%) and traditional Chinese medicine (54.9%)^8^. The incidence of clinically diagnosed ascites in patients with subacute liver failure is 60–80%, which is significantly more frequent than patients with acute liver failure^6,9–11^. Experimental animal models of acute liver failure, including surgical models and chemical models using hepatotoxins such as galactosamine and acetaminophen have been extensively investigated^12,13^. However, animal models of subacute liver failure have not yet been established. Reproducible animal model of subacute liver failure with ascites complication is needed.

Green tea made from the plant *Camellia sinensis L*. is one of the most popular beverages worldwide^14^. An increasing body of evidence demonstrates that green tea has health promotion effects, such as anti-inflammation, body weight reduction and alleviation of metabolic syndrome, and prevention of diabetes, cardiovascular diseases, cancer and neurodegenerative diseases^15^. The major functional components of green tea are catechins, including (-)-epigallocatechin-3-gallate (EGCG), (-)-epicatechin-3-gallate, (-)-epigallocatechin and (-)-epicatechin. EGCG accounts for over half of catechins in green tea and is the most biologically-active ingredient with strong antioxidant and prooxidant properties^15^. Green tea extracts, with EGCG being a predominant active component have been used as a dietary supplement for weight loss^16^. However, cases of hepatotoxicity, including liver injury and acute liver failure requiring liver transplantation have been reported^17–24^. Thus, a tolerable upper intake level of 300 or 338 mg EGCG/person/day for food supplements was recently proposed^25,26^.

In the past five years, our group has elucidated toxicological mechanisms of EGCG, uncovered agents that promote EGCG hepatotoxicity, and found protective agents against EGCG hepatotoxicity in mice^27–29^. In the present study, we step-wise elevated EGCG dose levels to induce hepatotoxicity. After one month of daily treatment with EGCG, to our surprise, we found that the mice manifested severe liver failure with large amount of ascites. This unexpected finding suggests that EGCG could be used to develop models of subacute liver failure with ascites complication in mice.

## Results

### Step-wise increased doses of EGCG caused subacute liver failure with ascites complication

**Figure 1:**
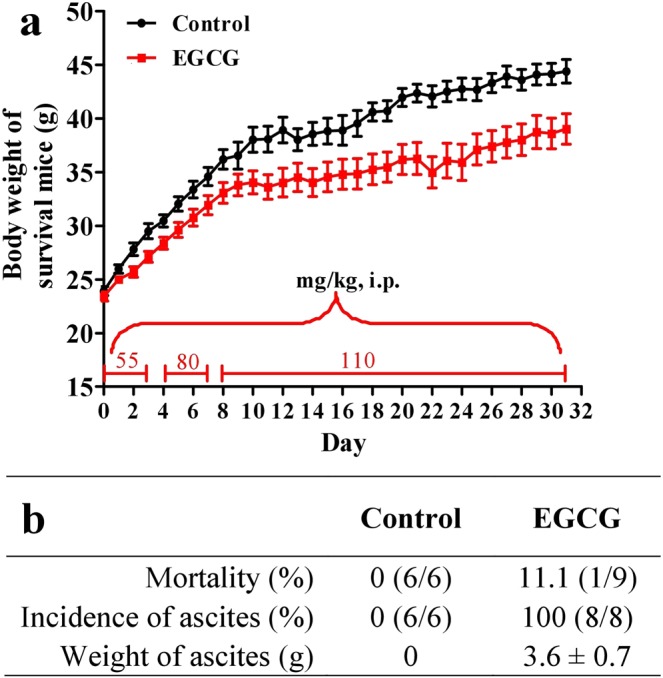
EGCG dosage schedule for preparing subacute liver failure with ascites complication. Mice were i.p. injected with saline (n = 6) as control or EGCG (n = 9) as indicated in a. (**a**) Body weight. (**b**) Mortality, incidence of ascites and weight of ascites. Data are presented as the mean ± SEM in a.

**Figure 2:**
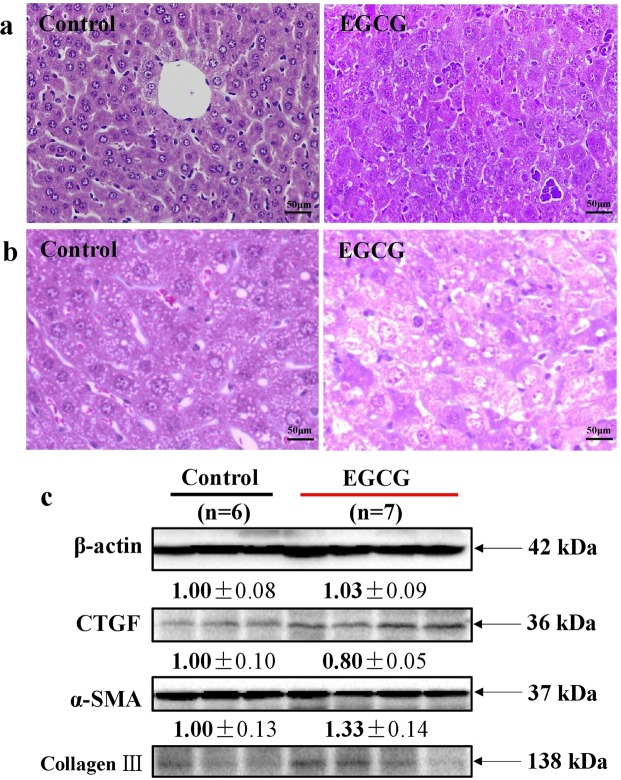
Histopathological observation and fibrosis-associated protein expression. Mice were treated according to the method shown in Fig. 1a. (**a**) H&E staining. (**b**) Masson staining. (**c**) Fibrosis-associated proteins. Full-length blots are presented in Supplementary Figure 1. Data are presented as the mean ± SEM in c.

**Figure 3:**
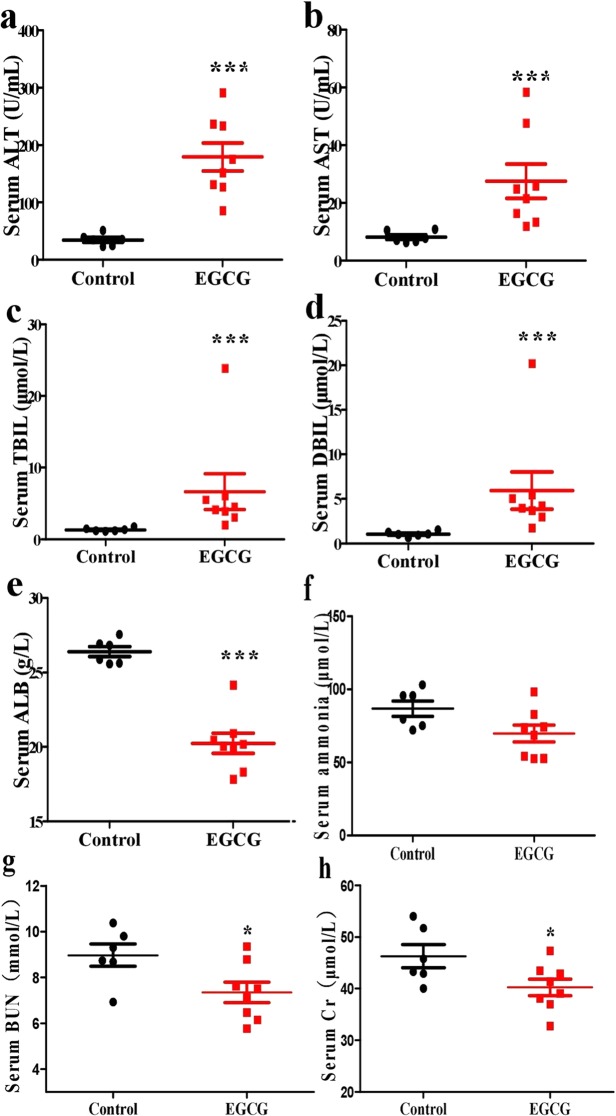
Influence of EGCG on serum biomarkers. Mice were treated according to the method shown in Fig. 1a. (**a**) ALT. (**b**) AST. (**c**) TBIL. (**d**) DBIL. (**e**) ALB. (**f**) Ammonia. (**g**) BUN. (**h**) Cr. Data are presented as the mean ± SEM (n = 6 and 8 in control and EGCG group, respectively). ***P < 0.001, as compared to the control.

The drug regimen as indicated in Fig. 1a resulted in the death of one mouse (11.1%) on day 26. All survival mice in the EGCG group bear noticeable ascites as high as 3.6 g in average when sacrificed on day 32 (Fig. 1b). Such a subacute administration of EGCG caused liver failure. Haematoxylin and eosin (H&E) stained liver tissue slices showed extensive hepatocyte necrosis (Fig. 2a). Masson staining of the liver tissue slices did not suggest the formation of liver fibrosis (Fig. 2b). The absence of liver fibrosis was corroborated by fibrosis-associated proteins including connective tissue growth factor (CTGF), alpha smooth muscle actin (α-SMA) and collagen type III (collagen III), which were not significantly increase by the EGCG treatment (Fig. 2c). The subacute administration of EGCG significantly increased serum alanine aminotransferase (ALT) (Fig. 3a), aspartate aminotransferase (AST) (Fig. 3b), total bilirubin (TBIL) (Fig. 3c) and direct bilirubin (DBIL) (Fig. 3d) as well as significantly decreased serum albumin (ALB) (Fig. 3e). Serum ammonia concentrations were not altered by EGCG treatments (Fig. 3f), suggesting that the EGCG treatments did not trigger encephalopathy. EGCG treatments did not result in kidney injury as evidenced by blood urea nitrogen (BUN) and serum creatinine (Cr) levels (Fig. 3g,h). Hepatic cytokine levels including IL-1β, IL-2, IL-6, IL-10, TNF-α and INF-γ were not increased by EGCG treatments (Table 1), suggesting that hepatic inflammation did not occur in this model.

**Table 1:** Hepatic cytokine levels^a^.

	Control	EGCG
IL-1β (pg/mg pro.)	868.0 ± 91.6	752.9 ± 46.5
IL-2 (pg/mg pro.)	38.4 ± 4.0	33.2 ± 3.5
IL-6 (ng/ mg pro.)	4.8 ± 0.4	4.2 ± 0.3
IL-10 (ng/mg pro.)	19.1 ± 1.8	16.0 ± 1.5
TNF-α (ng/mg pro.)	10.3 ± 1.6	8.3 ± 0.7
INF-γ (ng/mg pro.)	37.4 ± 4.4	36.3 ± 3.1

^a^Hepatic cytokine levels were measured using ELISA kits (BD Bioscience, San Jose, CA, USA) according to manufacture’s instructions.

### Step-wise increased doses of EGCG caused apoptotic response along with antioxidant adaptive response in the liver

**Figure 4:**
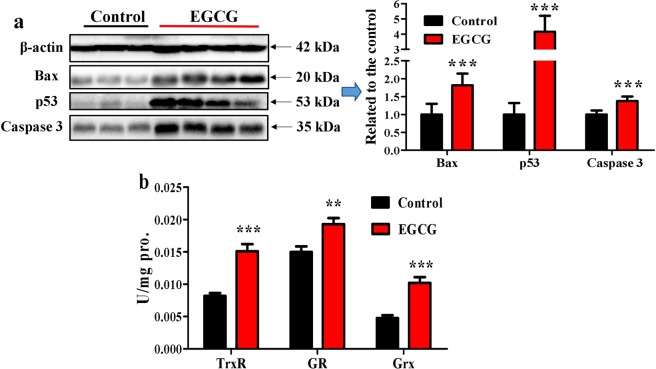
Influence of EGCG on hepatic apoptosis-associated proteins and activities of hepatic TrxR, GR and Grx. Mice were treated according to the method shown in Fig. 1a. (**a**) Hepatic apoptosis-associated proteins. (**b**) Hepatic TrxR, GR and Grx activities. Full-length blots are presented in Supplementary Fig. 2. Data are presented as the mean ± SEM (n = 6 and 8 in control and EGCG group, respectively). **P < 0.01 and ***P < 0.001, as compared to the control.

In the livers of EGCG-treated mice, there was elevated expression of apoptosis-associated proteins, such as Bax, p53 and caspase 3 (Fig. 4a). In addition, hepatic thioredoxin reductase (TrxR), glutathione reductase (GR) and glutaredoxin (Grx) activities were also increased (Fig. 4b), suggesting that the adaptive responses would make the mice tolerable to further EGCG treatments at otherwise lethal doses.

### Influence of step-wise increased doses of EGCG on urea transporter A1 and aquaporin 2 in the kidney and liver

**Figure 5:**
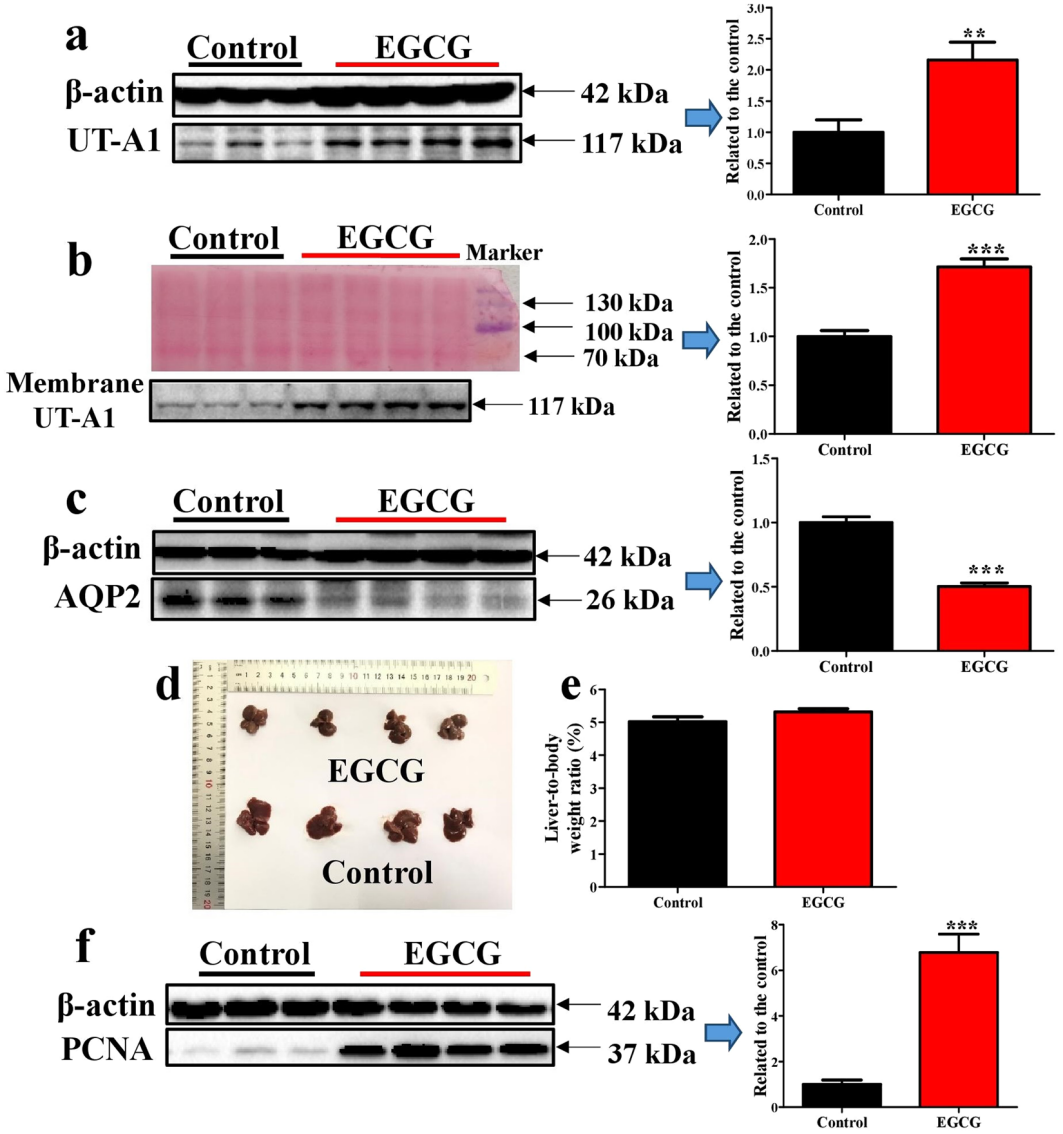
Influence of EGCG on renal UT-A1 and hepatic AQP2. Mice were treated according to the method shown in Fig. 1a. (**a**) Renal UT-A1. (**b**) Renal membrane UT-A1. (**c**) Hepatic AQP2. (**d**) Liver image. (**e**) Liver-to-body weight ratio. (**f**) Hepatic PCNA. All full-length blots are presented in Supplementary Fig. 3. Data are presented as the mean ± SEM (n = 6 in control group and n = 7 or 8 in EGCG group). **P < 0.01 and ***P < 0.001, as compared to the control.

To investigate the underlying mechanism leading to ascites formation, we determined the expression of water metabolism proteins. Renal urea transporter A1 (UT-A1) and renal membrane UT-A1 were significantly up-regulated by EGCG (Fig. 5a,b). The results suggest that EGCG promotes body water reabsorption by altering the expression of renal water metabolism-related proteins, leading to increased water retention and ascites formation. Aquaporin 2 (AQP2), a protein vital for water reabsorption, was significantly down-regulated in the livers of EGCG-treated mice (Fig. 5c), suggesting that the hepatocytes were in a state of low water content. This may account for the significantly lowered liver size in EGCG-treated mice (Fig. 5d). However, liver-to-body weight ratio did not change following EGCG treatments (Fig. 5e). Liver regeneration as indicated by proliferating cell nuclear antigen (PCNA) was enhanced by EGCG (Fig. 5f), possibly due to repairing of the damaged liver cells. It appears that the consequence of reduced live size could be either due to the lower water content of the hepatocytes or a general inhibitory effect of EGCG on growth reflected in body weight and liver weight.

## Discussion

The present study provides the first animal model of subacute liver failure with ascites complication. This model has a feature of low mortality (<12%), high incidence of ascites (100% in survival mice) and high ascites-body weight ratio (on average > 13%). These parameters are better than reported animal models of liver cirrhosis with ascites complication, which usually do not simultaneously exhibit the features of low mortality, high incidence of ascites and high ascites-body weight ratio. In general, when the mortality rates were controlled to be less than or equal to 20%, the ascites-body weight ratios are low (≤ 2%). When the ascites-body weight ratios are high (≥ 5%), the incidences of ascites are low (≤ 50%). For example, a rat model of liver cirrhosis induced by inhalation of carbon tetrachloride alone or the combination with phenobarbital resulted in 19–20% mortality, 65–80% incidence of ascites and 1.3–1.8% ascites-body weight ratio^30,31^. Mouse or rat models of liver cirrhosis generated via bile duct ligation may have an ascites-body weight ratio as high as 6.0%; however, the incidence of ascites was only 44–45%^32,33^.

Gimson *et al*. found that over half of the patients with subacute liver failure had small-sized liver^6^. In the present study, we also observed small-sized liver in most EGCG-treated mice. The consequence could be either due to the lower water content of the hepatocytes as indicated by reduced hepatic AQP2 or a general inhibitory effect of EGCG on growth reflected in body weight and liver weight since liver-to-body weight ratio did not change following EGCG treatments. It is known that liver cirrhosis is associated with abnormal renal water retention^34^. In patients with liver cirrhosis, impaired renal water excretion is involved in the pathogenesis of ascites formation^35^. AQP2 expression and its trafficking to plasma membrane play an important role in renal water handling. The mRNA expression of renal aquaporin positively correlated with the volume of ascites and the trafficking of AQP2 to plasma membrane of renal collecting duct was increased in cirrhotic rats^35,36^. Renal urea transporter UT-A1 also participates in renal water handing^37^. We did not detected significant alterations of renal AQP2 in the model mice, but found that renal UT-A1 and renal membrane UT-A1 were significantly increased by EGCG. This result implies that certain UT-A1 inhibitor^38–40^ would be useful to reduce ascites in patients with subacute liver failure.

Polyphenolic compounds are known to have antioxidant or prooxidant activities^41–43^. Seventy years ago, Korpassy and Kovacs reported a liver cirrhosis model produced by tannic acid in rats^44^. Like tannic acid, EGCG is a potent redox-active polyphenol possesses both antioxidant and prooxidant properties, depending upon dose levels and exposure environments^28,45^. Thus, EGCG is able to protect the liver from injury induced by hepatotoxins such as carbon tetrachloride in mice^46,47^ or can cause liver injury and acute liver failure in mice and humans at high-dose levels^17–24,27,29,48^. Therefore, it is not surprising that EGCG can be used to prepare an animal model of subacute liver failure as shown in the present study. However, our experience shows that a successful model of subacute liver failure with ascites complication needs careful considerations of dose elevation regimen. EGCG at pharmacological dose (45 mg/kg, i.p.) that approaches to toxicological doses (55 mg/kg, i.p.) in mice increased hepatic thioredoxin reductase 1^27,45^, moreover, EGCG at 55 mg/kg (i.p.) that normally results in hepatotoxicity in mice markedly activated hepatic Nrf2 defense system^27,29^. In the present study, while EGCG caused liver failure, hepatic TrxR, GR and Grx activities were consistently increased. The adaptive responses of the liver to EGCG at pharmacological and toxicological dose levels in essence allow mice to be tolerable to further EGCG treatments at otherwise lethal doses. This concept is compatible to a recent study, wherein James *et al*. found that precondition of mice with EGCG at a pharmacological dose could effectively attenuated liver injury induced by subsequent administration of EGCG at a toxic dose^49^. Concerning mouse model of subacute liver failure with ascites complication, it is desirable to have low mortality, high incidence of ascites and noticeable ascitic fluid. Our experience suggests that step-wise increase of EGCG can reach this goal. In the present study, mortality was 11.1%, i.e., one mouse out of 9 mice subjected to the step-wise increased doses of EGCG treatment died on day 26. We measured serum BUN and Cr and found that EGCG did not increase these parameters, suggesting that mortality was not associated with kidney damage. The cause of the death is unclear. It is possible that hepatic adaptive response had not been effectively induced by the stepwise EGCG treatment in the dead mouse.

Green tea extracts, with EGCG as a major constituent, are alluded to have many beneficial health effects, including liver-protective function^46,47^. Without knowing the risk of liver injury, consumers of green tea extracts may take more when they feel uncomfortable with an expectation to improve the symptoms. The present study suggests that such a behavior probably leads to a vicious cycle, resulting in a consequence of severe livery injury or liver failure.

In conclusion, step-wise elevated doses of EGCG in mice can result in liver failure, which exhibits some of the features observed in patients with subacute liver failure, particularly with ascites. This animal model hence should be a useful tool for studying the pathogenesis of subacute liver failure with ascites complication and would be helpful for uncovering preventive agent as well as more effective therapy for subacute liver failure.

## Materials and Methods

### Chemicals and drugs

EGCG (>99%) purified from green tea was obtained from Ebeikar Tea & Extracts Co., Ltd. (Hangzhou, China). Glutathione, oxidized glutathione, GR (from baker yeast), and nicotinamide adenine dinucleotide phosphate (NADPH) were all obtained from Sigma (St. Louis, MO, USA). RIPA regent and BCA protein assay kit were products of Beyotime Biotechnology (Shanghai, China). Anti-mouse and anti-rabbit secondary antibodies were obtained from Santa Cruz (Dallas, TX, USA). The primary antibodies against UT-A1 (SAB5200108) and β-actin (A5441) were purchased from Sigma (St. Louis, MO, USA). The primary antibodies against AQP2 (#3487), Bax (#2772), p53 (#9282) and caspase3 (#9662) were purchased from Cell Signal Technology, Inc. (Boston, MA, USA). The primary antibodies against CTGF (ab6992), α-SMA (ab7817) and collagen III (ab7778) were products of Abcam (Cambridge, UK). The primary antibody against PCNA (BM0104) was purchased from Boster Biological Technology Co., Ltd. (Wuhan, China). ECL Plus reagent and PVDF membrane were purchased from Bio-Rad Laboratories, Inc. (Hercules, CA, USA). Other chemicals were of the highest grade available.

### Animals and treatments

Male Kunming mice (22–25 g) and their diet were purchased from Shanghai SLAC Laboratory Animal Co., Ltd. (Shanghai, China). The mice were given free access to food and water and housed in a room with temperature of 25 ± 1 °C, relative humidity of 50 ± 10%, and a 12 h light/dark cycles. All animals received humane care in compliance with the Guide for the Care and Use of Laboratory Animals formulated by the Ministry of Science and Technology of the People′s Republic of China. Experimental animal protocols were approved by the Ethics Committee of Anhui Agricultural University. Fifteen mice were randomly divided into two groups, and were intraperitoneally (i.p.) injected daily with saline as control (n = 6) or EGCG (n = 9). The injected dose of EGCG was step-wisely increased: 55 mg/kg from day 0 to day 3, 80 mg/kg from day 4 to day 7, and 110 mg/kg from day 8 to day 31. At 24 h post the last treatment, mice were sacrificed. Laparotomy was performed and ascites weight was evaluated by the difference in body weight of a mouse before and after removing ascitic fluid. Peripheral blood from the ophthalmic veins was collected into an eppendorf tube. The tube was centrifuged at 9,000 g at 4 °C for 10 min to obtain serum. The liver and kidney tissues were collected.

### Serum biomarkers

Serum ALT, AST, ALB, TBIL, DBIL, BUN, Cr and ammonia levels were measured with commercial kits purchased from the Nanjing Jiancheng Bioengineering Institute (Nanjing, China).

### Histopathological observation

Liver samples were excised, fixed in 10% formalin and embedded in paraffin. The sections were stained with H&E or Masson, and observed under a light microscopy.

### Western-blotting analysis

The concentrations of total protein extracted with the RIPA reagent were detected by the BCA protein assay kit. Protein extracts were diluted, boiled with SDS PAGE loading buffer at 95 °C for 10 min and then were subjected to protein separation by loading onto 10% SDS PAGE. Proteins in the gel were transferred to a PVDF membrane. The membranes were blocked with 5% skimmed milk powder in tris buffered saline with 0.05% Tween 20 (TBS-T) for 2 h at room temperature. After a short wash with TBS-T, the membranes were incubated with specific primary antibodies which were diluted in TBS-T by 200 to 5,000 folds overnight at 4 °C. The membranes were washed four times with TBS-T and then were incubated with secondary antibody diluted in TBS-T by 2500- or 5000-fold for 1 h at room temperature. After washing four times with TBST, the detection of proteins was performed using the ChemiDoc XRS + detection system (ECL, Bio-Rad). The immunoblots were analyzed with the Quantity One^®^ Image Analyzer software program (Bio-Rad). β-actin was used as an internal reference for examined proteins except membrane UT-A1. Equal loading of protein amounts while evaluating membrane UT-A1 was demonstrated using Ponceau staining.

### Measurement of enzymatic activity

Liver tissue was homogenized in ice cold 150 mM phosphate buffer solution, pH 7.2, containing 1 mM EDTANa_2_. The resultant homogenate was centrifuged at 15,000 g at 4 °C for 15 min to obtain supernatant. Protein concentration was determined by the Bradford dye-binding assay with bovine serum albumin as a standard. TrxR activity was determined using NADPH dependent 5,5′-dithio-bis(2-nitrobenzoic acid) reduction method, described by Smith *et al*. with some modifications^50,51^. TrxR activity was calculated by subtracting the slope rate of the reaction in the presence of auranofin from the slope rate of the reaction in the absence of auranofin. GR activity was measured according to the method of Carlberg *et al*. with oxidized glutathione as a substrate^52^. Grx activity was determined by the method of Scian *et al*. with 2-hydroxyethyl disulfide as a model substrate^45,53,54^. The enzymatic activities were presented as nmol of NADPH oxidized/min/mg protein.

### Preparation of renal membrane fractions

Renal membrane fraction was prepared according to the method described by Tiwari *et al*.^55,56^ with some modifications. Briefly, kidney tissue was homogenized in chilled isolation solution (250 mM sucrose, 10 mM triethanolamine, 1 µg/mL leupeptin, and 0.1 µg/mL phenylmethylsulfonyl fluoride, pH 7.6). The homogenate was frozen at −80 °C overnight. The thawed homogenate was centrifuged at 17,000 g for 20 min at 4 °C. The resultant precipitate was homogenized in the isolation solution. After centrifugation at 17,000 for 20 min, the precipitate was stored at −80 °C and was resuspended in the isolation solution prior to Western-blotting analysis.

### Measurement of hepatic cytokine levels

Hepatic IL-1β, IL-2, IL-6, IL-10, TNF-α and INF-γ levels were measured using ELISA kits (BD Bioscience, San Jose, CA, USA) according to manufacture′s instructions.

### Statistical analysis

All data are presented as mean ± standard error of the mean (SEM). The difference between two groups was examined by Student′s t-test. Two-way analysis of variance (ANOVA) was used for comparison of body weight of two groups over entire experimental period. All statistical analyses were performed using GraphPad software (Prism version 5, San Diego, CA, USA). A P-value less than 0.05 was considered to be statistically significant.

## Supplementary information

Supplementary Information

## Data Availability

The datasets generated during and/or analysed during the current study are available from the corresponding author on reasonable request.
