# Supplementary material for: A mouse model of subacute liver failure with ascites induced by step-wise increased doses of (-)-epigallocatechin-3-gallate
Source: Sci Rep. 2019 Dec 2;9:18102. doi: 10.1038/s41598-019-54691-0 (PMC6888815; doi:10.1038/s41598-019-54691-0)
Supplement: Supplementary file 1 — Supplementary Information [file 41598_2019_54691_MOESM1_ESM.docx]

**Supplementary Information**

**A mouse model of subacute liver failure with ascites induced by step-wise increased doses of (-)-epigallocatechin-3-gallate**

Xiaoxiao Wang^a^, Lumin Yang^a^, Jiajia Wang^a^, Yafei Zhang^b, c^, Ruixia Dong^d^, Ximing Wu^a^, Chung S. Yang^e, f^, Zhenhua Zhang^b, c^ *, Jinsong Zhang^a, f^ *

^a^ State Key Laboratory of Tea Plant Biology and Utilization, School of Tea & Food Science, Anhui Agricultural University, Hefei, Anhui, China

^b^ Department of Infectious Diseases, The Second Affiliated Hospital, Anhui Medical University, Hefei, Anhui, China

^c^ School of Pharmacy, Anhui Medical University, Hefei, Anhui, P.R. China

^d^ Department of Forestry and Technology, Lishui Vocational and Technical College, Lishui, Zhejiang, China

^e^ Department of Chemical Biology, Ernest Mario School of Pharmacy, Rutgers, The State University of New Jersey, Piscataway, NJ, USA

^f^ International Joint Research Laboratory of Tea Chemistry and Health Effects, Anhui Agricultural University, Hefei, Anhui, China


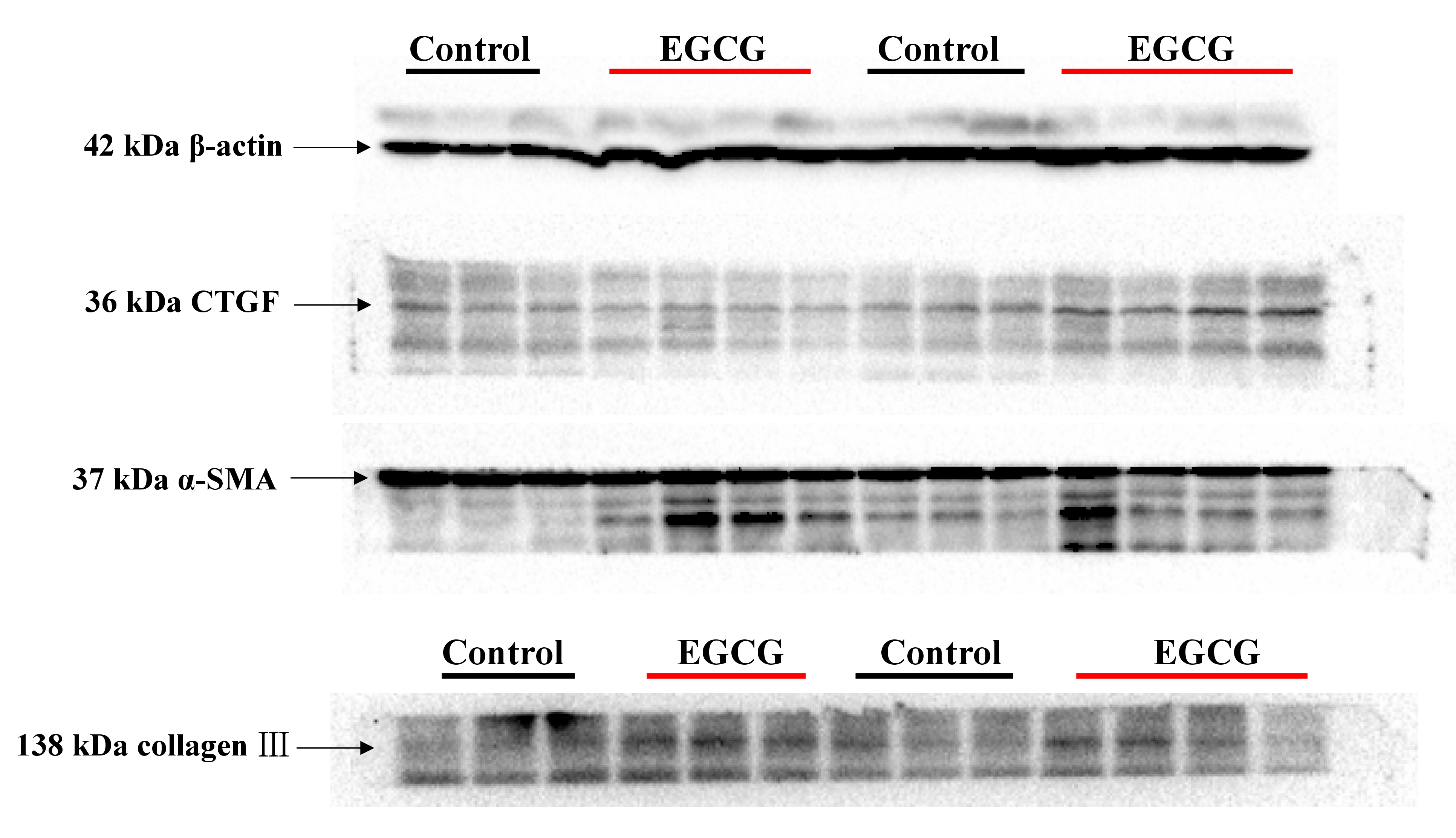


**Supplementary Figure 1. Non-cropped blots for the experiment shown in Fig. 2c of the main article.**


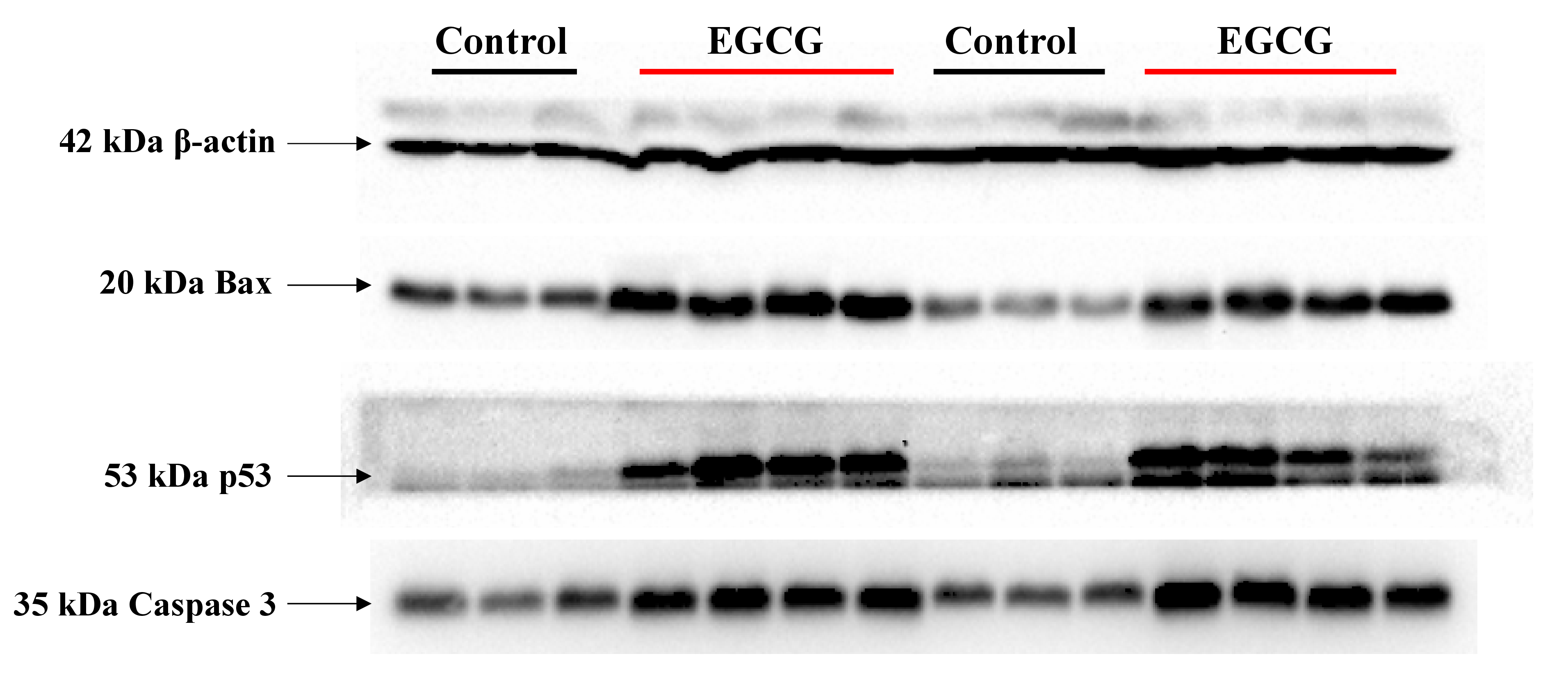


**Supplementary Figure 2. Non-cropped blots for the experiment shown in Fig. 4a of the main article.**


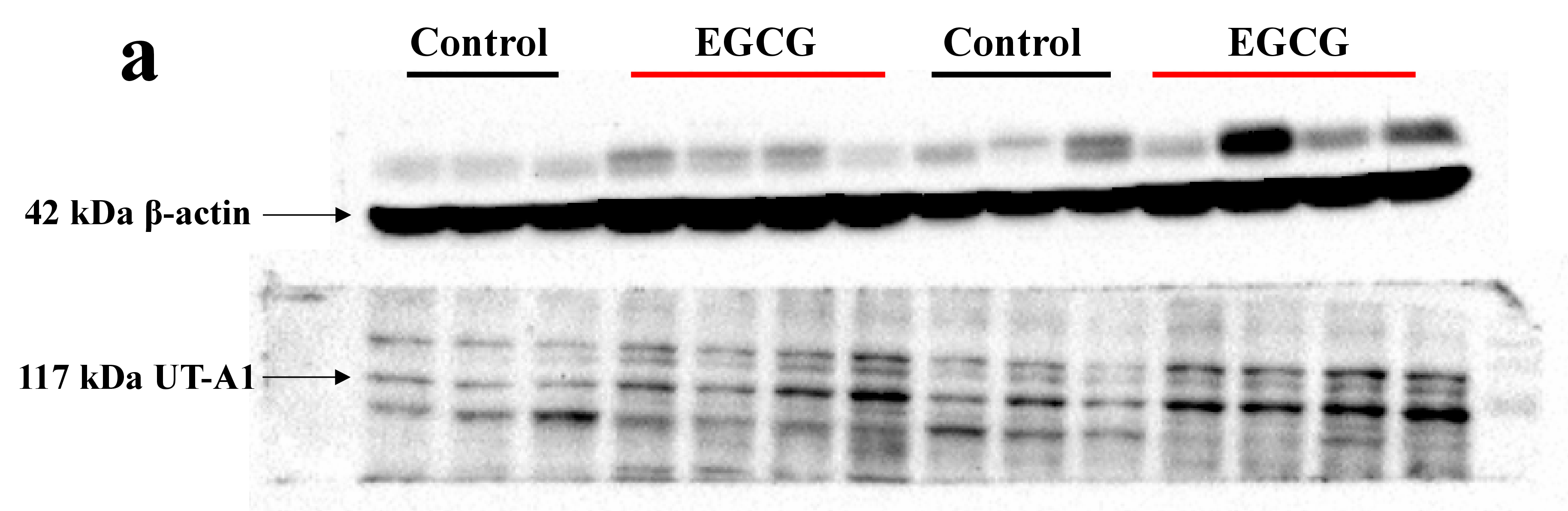

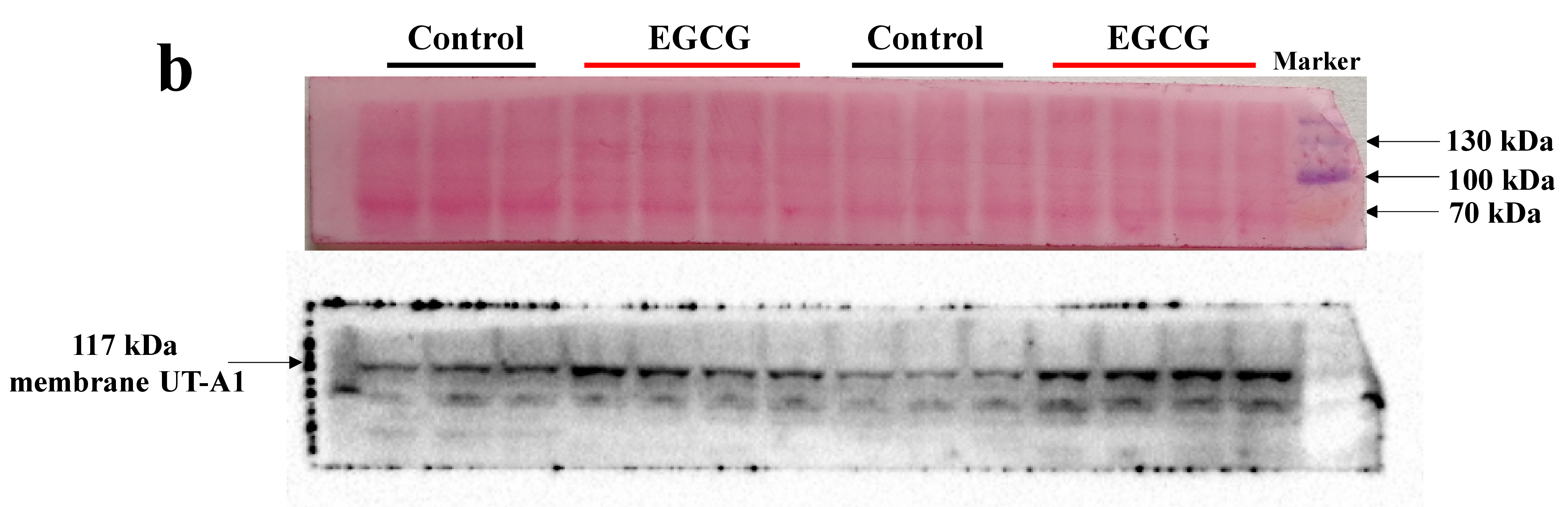

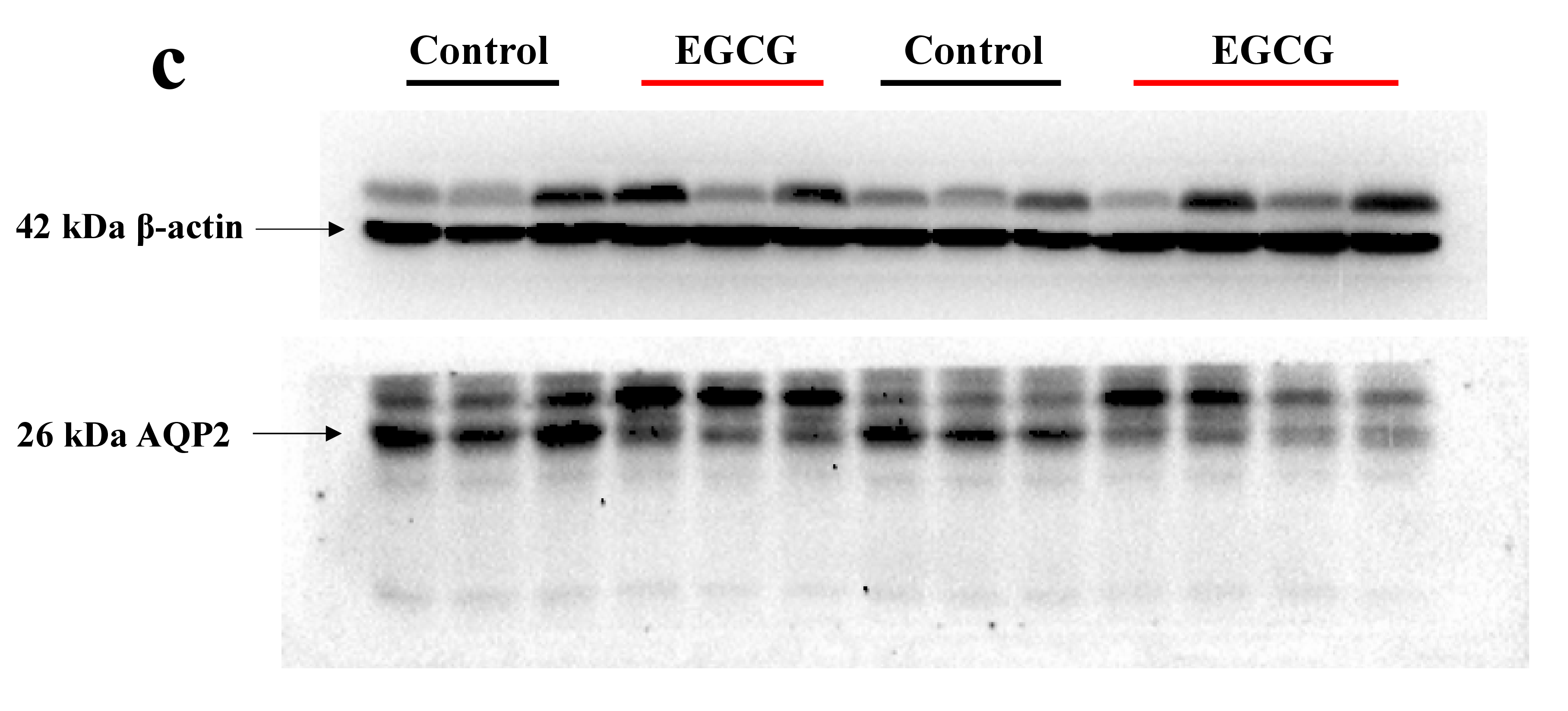

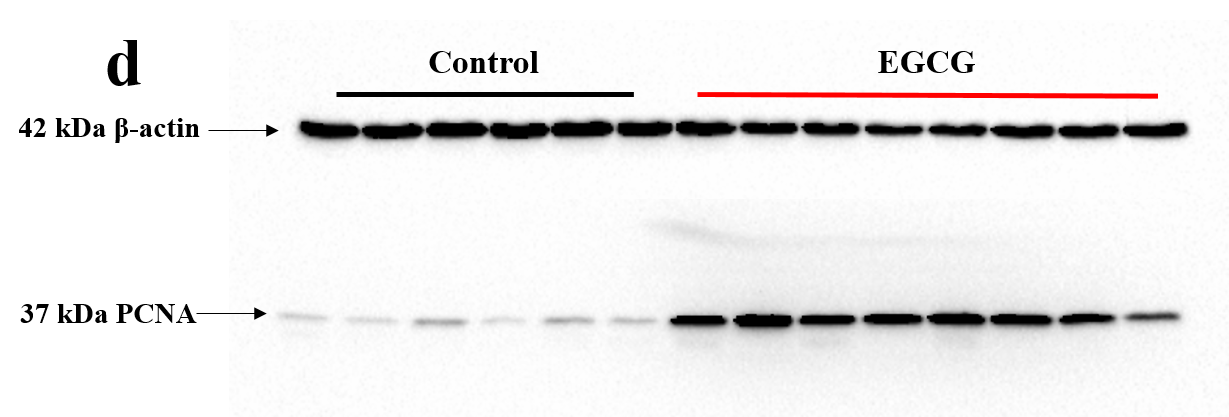


**Supplementary Figure 3. Non-cropped blot for the experiment shown in Fig. 5 of the main article.** (a), (b), (c) and (d) correspond to Fig. 5a, 5b, 5c and 5f of the main article, respectively.
